# Genomic relevance of *FGF14* and associated genes on the prognosis of pancreatic cancer

**DOI:** 10.1371/journal.pone.0252344

**Published:** 2021-06-01

**Authors:** Aroosha Raja, Muhammad Faraz Arshad Malik, Farhan Haq

**Affiliations:** COMSATS University, Islamabad, Pakistan; Wayne State University, UNITED STATES

## Abstract

**Background:**

Fibroblast (FGFs) and insulin (IGF) growth factor pathways are among 10 most recurrently altered genomic pathways in pancreatic ductal adenocarcinoma (PDAC). However, the prognostic and therapeutic relevance of *FGF* and *IGF* pathways in PDAC is largely unknown.

**Methods:**

We investigated the relationship between fibroblast and insulin pathway gene expression and clinicopathological features in three independent transcriptomic cohorts of 532 PDAC patients. Furthermore, we have examined the coexpressed genes specific to the prognostic marker identified from these cohorts. Statistical tests including Fisher-exact\Chi-square, Kaplan–Meier, Pearson Correlation and cox regression analyses were performed. Additionally, pathway analysis of gene-specific co-expressed genes was also performed.

**Results:**

The dysregulation of six genes including *FGF9*, *FGF14*, *FGFR1*, *FGFR4*, *IGF2BP2* and *IGF2BP3* were significantly associated with different clinical characteristics (including grade, stage, recurrence and nodes) in PDAC cohorts. 11 genes (including *FGF9*, *FGF13*, *FGF14*, *FGF17*, *FGFR1*, *FGFRL1*, *FGFBP3*, *IGFBP3*, *IGF2BP2*, *IGF2BP3* and *IGFBPL1*) showed association with overall survival in different PDAC cohorts. Interestingly, overexpression of *FGF14* was found associated with better overall survival (OS) in all three cohorts. Of note, multivariate analysis also revealed *FGF14* as an independent prognostic marker for better OS in all three cohorts. Furthermore, *FMN2* and *PGR* were among the top genes that correlated with *FGF14* in all 3 cohorts. Of note, overexpression of *FMN2* and *PGR* was found significantly associated with good overall survival in PDAC patients, suggesting *FMN2* and *PGR* can also act as potential markers for the prediction of prognosis in PDAC patients.

**Conclusion:**

*FGF14* may define a distinct subset of PDAC patients with better prognosis. Moreover, *FGF14*-based sub-classification of PDAC suggests that *FMN2* and *PGR* can be employed as good prognostic markers in PDAC and this classification may lead to new therapeutic approaches.

## Introduction

Pancreatic adenocarcinoma (PDAC) is the fourth most fatal cancer with increasing mortality rate [1]. Interestingly, PDAC accounts for more than 95% of exocrine pancreatic cancers [2]. PDAC imposes great mortality risk due to its rapid spread, poor prognosis, scarcity of available treatment options, late detection, invulnerability towards chemotherapy and lower overall survival rate [3]. The sensitivity profiles exhibited by PDAC towards chemotherapy and radiotherapy remain very low [4, 5]. As PDAC shows very high resistance to treatment, it is ranked among the most aggressive tumor types [6]. The major non-genetic risk factors of PDAC are chronic pancreatitis, diabetes, and obesity [7]. Among all these risk factors, diabetes is the most prominent factor, as pancreatic cancer and diabetes both affect the same organ [8]. In addition, both of these diseases also share common risk factors [9]. Keeping in view the aggressive nature and low therapeutic options available for PDAC, proper screening and identification of suitable therapeutic targets is urgently required [10].

The aggressiveness exhibited by PDAC is not only due to environmental or specific molecular traits but also due to genetically altered pathways [11]. Importantly, two growth factor receptor pathways including Fibroblast growth factors (FGFs) and insulin growth factors (IGF) are frequently reported in PDAC [12]. For instance, altered expression of *FGFR1* and *FGFR2* receptors is reported in PDAC pathogenesis [13, 14]. Very importantly, Motoda et al. revealed that *FGF19* (specific ligand of *FGFR4*) is involved in suppressing PDAC progression by stimulating *FGFR4* expression i.e. the overexpression of *FGFR4* correlates with good survival outcomes in PDAC [15]. Furthermore, studies also reported that dysregulation of genes of Insulin growth factor pathway also lead to excessive growth stimulation in human pancreatic cancer [16–18]. Particularly, *IGF-I* is considered as potential therapeutic target for pancreatic tumors (along with *IGF-IR*) [19].

In this study, we aimed to investigate integrated role of FGFs and IGF family genes on the prognosis of PDAC patients. In addition, we also examined gene-specific co-expressed genes with the independent prognostic marker revealed in the first half of analyses and investigated their significance in PDAC patients. For these purposes, microarray and RNA-seq data of 532 PDAC patients were analyzed in PDAC patients.

## Materials and methods

### Data collection and processing

The overall study design is presented in **S1 Fig in** S1 File. This study is certified and approved by **COMSATS University review board (CIIT/Bio/ERB/16/21)**. Transcriptomic profiling of *FGF* and *IGF* pathway genes in 532 (65+179+288) PDAC patients was performed. The discovery cohort 1 (GSE62452) was retrieved from Gene Expression Omnibus (GEO) database [20]. Robust multiarray average (RMA) normalization was performed to generate mRNA expression. The expression data was divided into high and low expression groups based on their median values. The clinicopathological parameters of cohort 1 included grade, stage and survival status, details are summarized in **S1 Table in** S1 File. The discovery cohort 2 of 179 PDAC patients was obtained from public cBioPortal database (https://www.cbioportal.org/). Similarly, reads per kilobase million (RPKM) expression values were divided into high and low expression groups. The clinicopathological parameters of cohort 2 included age, gender, stage, grade, T-stage, nodes, metastasis, residual, survival and disease-free survival (DFS) status, type-2 diabetes and alcohol consumption history (**S2 Table in** S1 File). For validation of PDAC results, expression data of 288 PDAC patients was retrieved from ArrayExpress (E-MTAB-6134) database and RMA processing was done to generate mRNA expression. The cohort 3 included classification system based on tumor features and includes clinicopathological parameters such as gender, nodes, survival status and t-stage. The details of clinicopathological features of cohort 3 are described in **S3 Table in** S1 File. R-script was used for the modification and pre-processing of all the cohorts under study.

### Functional analysis of gene-specific coexpressed genes

The gene-specific coexpressed genes obtained through the analysis were subjected to pathway analysis using the ClusterProfiler program [21] implemented in R software version 4.0.3. Only genes that were common in cohort 1, 2 and 3 were subjected to this analysis. The analysis was conducted under the specific parameters including statistical Fisher’s exact test, along with Benjamini correction and the cut-off value set for the analysis was adjusted P-value <0.05.

### Statistical analysis

IBM SPSS^®^ software version 20.0 (Armonk, NY, USA) and R software version 4.0.3 were used for the statistical evaluation of the data. Statistical correlation of gene expression was performed against clinical parameters available for each cohort. The association was determined using Chi-square and Fisher Exact tests. We generated Kaplan Meier plots to determine the association with overall survival and disease-free survival. Furthermore, to predict independent prognostic markers for PDAC, cox regression univariate and multivariate analysis was performed. The probability value less than 0.05 was considered statistically significant. To examine gene-specific coexpressed genes, Pearson’s correlation using the gene of interest was employed on all the three cohorts of PDAC patients using R-script. VennDiagram [22] package implemented in R was used to generate customizable venn diagrams to identify common significantly associated genes among the cohorts used in the analysis.

## Results

### Association of genes with clinical parameters in cohort 1

The dichotomized expression values of FGF and IGF pathway genes were evaluated against the clinical features available. According to the results, most of the genes showed significant (*p ≤ 0.05) association with early tumor grade and stage. The information regarding significant association of genes with clinical parameters is summarized in **S4 Table in** S1 File. The overexpression of *FGF6*, *FGF9*, *FGF14*, *FGFR1* and *FGFR4* genes was correlated with early tumor grade (i.e., grade stage < = 2) **(S4 Table in** S1 File**)**. In addition, overexpression of *FGFR1* and *IGFBP4* genes was found significantly associated with early tumor stage (i.e., tumor stage < = 2). However, the overexpression of *FGFR1OP2*, *IGF2BP2*, *IGF2BP3*, *IGFL2* and *IRS1* genes was significant in advance stages of grade (**S4 Table in** S1 File).

**Fig 1:**
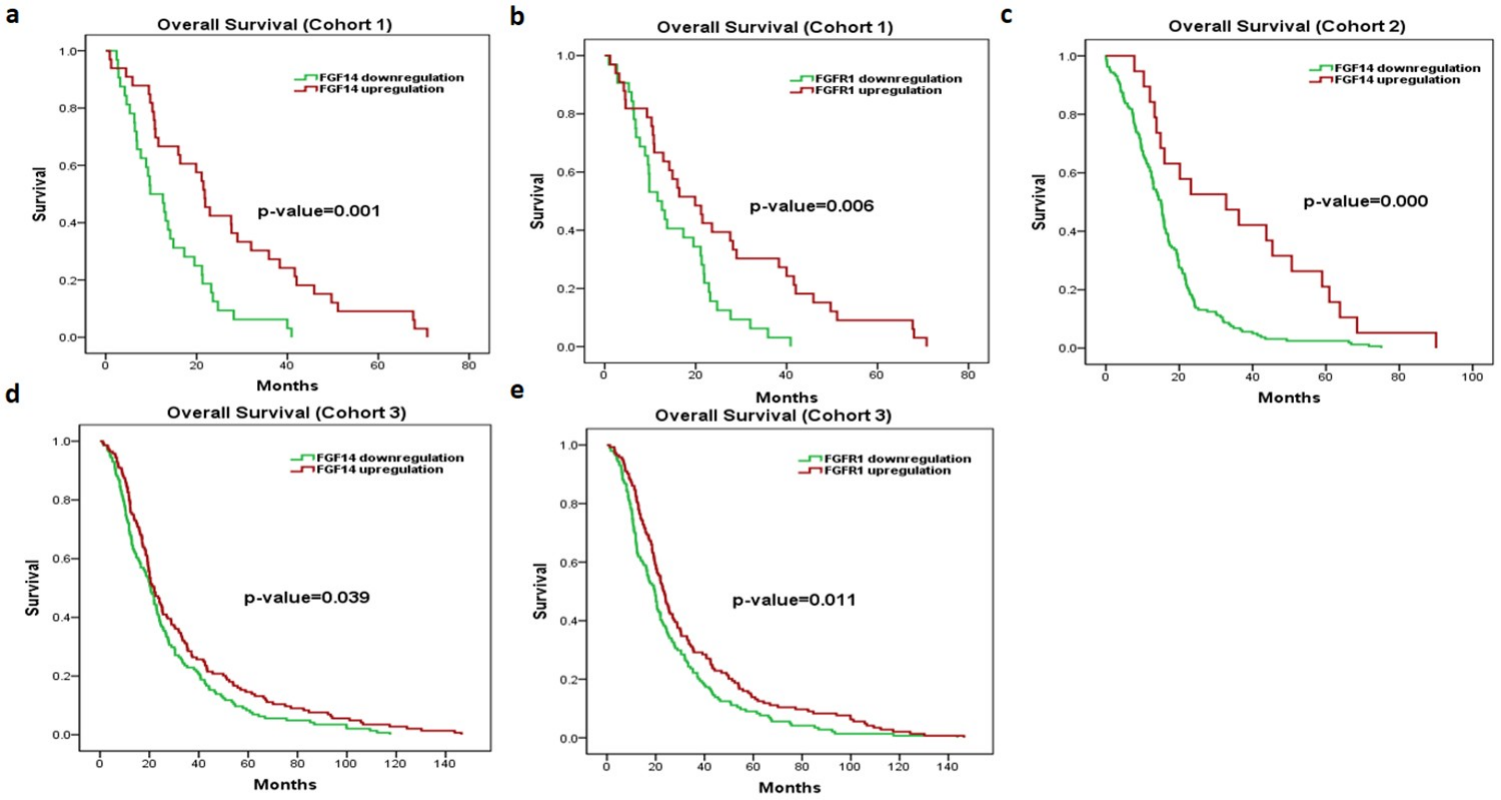
Kaplan–Meier survival analyses of *FGF14* and *FGFR1* in PDAC cohorts 1, 2 and 3. Red represents expression ≥ the median, while green represents expression < the median. Overall survival plots of *FGF14* (**a**), *FGFR1* (**b**) in cohort 1. Overall survival plots of *FGF14* (**c**) in cohort 2. Overall survival plots of *FGF14* (**d**), *FGFR1* (**e**) in cohort 3. (* p ≤ 0.05).

Kaplan-Meier survival analysis showed significantly lower overall survival (OS) of *IGF2BP2* gene and *IGF2BP3* overexpression, with Mantel-cox p-value of 0.046 and 0.021, respectively. (**S2 Fig in** S1 File) Interestingly, the overexpression of *FGFR1* and *FGF14* genes showed good overall survival (OS), with Mantel-cox p-value of 0.006 and 0.001, respectively (Fig 1).

Next, univariate/multivariate cox regression survival analysis was performed to identify potential prognostic markers for overall survival. According to univariate results, overall survival was significantly associated with *FGF14* (HR 0.398, 95% CI 0.232–0.682, P = 0.001), *FGFR1* (HR 0.475, 95% CI 0.277–0.817, P = 0.007), *IGF2BP2* (HR 1.667, 95% CI 1.003–2.771, P = 0.049), *IGF2BP3* (HR 1.836, 95% CI 1.089–3.094, P = 0.023) and Grade (HR 0.504, 95% CI 0.295–0.859, P = 0.012) (Table 1). Of note, multivariate analysis revealed *FGF14* gene as the only independent prognostic marker for better overall survival (i.e., HR < 1) of PDAC patients (Table 1).

**Table 1:** Univariate and multivariate analysis of genes for overall survival in cohort 1.

Factors	Univariate Analysis				Multivariable analysis			
	P-value	HR	95.0% CI		P-value		95.0% CI	
			Lower	Upper		HR	Lower	Upper
FGF14	**0.001**	**0.398**	**0.232**	**0.682**	**0.045**	**0.545**	**0.301**	**0.987**
FGFR1	**0.007**	0.475	0.277	0.817				
IGF2BP2	**0.049**	1.667	1.003	2.771				
IGF2BP3	**0.023**	1.836	1.089	3.094				
Grade	**0.012**	0.504	0.295	0.859				

### Association of genes with clinical parameters in cohort 2

In cohort 2, several genes under study have shown significant (*p ≤ 0.05) association with multiple clinicopathological features including age, gender, grade, stage, tumor recurrence, residual, nodes, survival, DFS status, diabetes and alcohol consumption history. The detailed information of significant association of genes with clinicopathological parameters is summarized in **S5 Table in** S1 File. For instance, overexpression of *FGF4* and *IGF2BP3* genes was associated with age < 50. The overexpression of *IRS2* genes was significantly associated with alcoholic PDAC patients. Interestingly, overexpression of *FGFR2*, *FGF7* genes and low expression of *FGFR4* gene was found significantly associated with diabetic PDAC patients. The overexpression *FGF7*, *FGF9*, *FGF14*, *FGF18* and *IGF1* genes was found significant in early tumor grades. However, only overexpression of *FGFBP1* gene showed association with advanced tumor grade. Furthermore, overexpression of *FGF12*, *FGF14*, *FGF17*, *FGFR1*, *FGFBP3* and *IGFBPL1* genes was found in early tumor stage, while, overexpression of *IGF1R*, *IGF2BP2* and *INSRR* was found in advanced tumor stages. In addition, overexpression of *FGF1*, *FGF9* and *FGF14* genes were found significant in node negative status of PDAC patients (**S5 Table in** S1 File).

According to Kaplan Meier plots for overall survival, the overexpression of *FGFRL1* (p-value = 0.033), *IGFBP3* (p-value = 0.037) and *IGFL1* (p-value = 0.001) showed poor overall survival, while, overexpression of *FGF9* (p-value = 0.007), *FGF13* (p-value = 0.015), *FGF17* (p-value = 0.000), *FGFBP3* (p-value = 0.000) and *IGFBPL1* (p-value = 0.002) genes was associated with good overall survival of PDAC patients (**S3 Fig in** S1 File). Of note, *FGF14* (p-value = 0.000) in overexpressed state correlated with good OS of PDAC patients (Fig 1). According to Kaplan Meier plots for disease-free survival (DFS), overexpression of *FGF9* (p-value = 0.012), *FGF12* (p-value = 0.001), *FGF13* (p-value = 0.014), *FGF14* (p-value = 0.000), *FGF17* (p-value = 0.000), *FGFBP3* (p-value = 0.000) and *IGFBPL1* (p-value = 0.000) genes showed increased DFS, while, overexpression of *FGF10* (p-value = 0.018), *FGFRL1* (p-value = 0.031) and *IGFL1* (p-value = 0.001) genes reduced DFS of PDAC patients (**S4 Fig in** S1 File).

Moreover, univariate analysis of discovery cohort 2 showed significant association of *FGF9* (HR 0.541, 95% CI 0.342–0.855, P = 0.008), *FGF13* (HR 0.510, 95% CI 0.293–0.887, P = 0.017), *FGF14* (HR 0.397, 95% CI 0.240–0.657, P = 0.000), *FGF17* (HR 0.244, 95% CI 0.106–0.560, P = 0.001), *FGFBP3* (HR 0.272, 95% CI 0.137–0.540, P = 0.000), *FGFRL1* (HR 1.658, 95% CI 1.035–2.656, P = 0.035), *IGFBP3* (HR 1.558, 95% CI 1.022–2.375, P = 0.039), *IGFBPL1* (HR 0.235, 95% CI 0.085–0.646, P = 0.005), Nodes (HR 0.714, 95% CI 0.513–0.995, P = 0.047) and Residual tumor (HR 0.673, 95% CI 0.486–0.932, P = 0.017) with hazard ratio and 95% CI presented in Table 2. Consistent with the results in cohort 1, *FGF14* (HR 0.531, 95% CI 0.303–0.930, P = 0.027) was found as independent prognostic marker in multivariate analysis.

**Table 2:** Univariate and multivariate analysis of genes for overall survival in cohort 2.

Factors	Univariate Analysis				Multivariable analysis			
	P-value	HR	95.0% CI		P-value	HR	95.0% CI	
			Lower	Upper			Lower	Upper
FGF9	**0.008**	0.541	0.342	0.855				
FGF13	**0.017**	0.510	0.293	0.887				
FGF14	**0.000**	**0.397**	**0.240**	**0.657**	**0.027**	**0.531**	**0.303**	**0.930**
FGF17	**0.001**	0.244	0.106	0.560				
FGFBP3	**0.000**	0.272	0.137	0.540				
FGFRL1	**0.035**	1.658	1.035	2.656				
IGFBP3	**0.039**	1.558	1.022	2.375				
IGFBPL1	**0.005**	0.235	0.085	0.646				
Nodes	**0.047**	0.714	0.513	0.995				
Residual	**0.017**	**0.673**	**0.486**	**0.932**	**0.014**	**0.654**	**0.466**	**0.918**

Of note, we have also evaluated the association of independent prognostic marker *FGF14* with IGF pathway genes. Interestingly, *FGF14* inversely correlated with important IGF pathway genes i.e. *IGF2BP1*, *IGF2BP2*, *IGF2BP3*, *IGFBP6*, *IGFL1*, *IGFL2* and *IGFL3* in cohort 1 and 2 of PDAC patients (**S6 Fig in** S1 File).

### Validation of *FGF14* as independent prognostic marker of PDAC

Interestingly, univariate analysis of validation cohort also showed significant association of *FGF14* (HR 0.782, 95% CI 0.619–0.988, P = 0.039), *FGFR1* (HR 0.740, 95% CI 0.586–0.935, P = 0.000) and nodes (HR 1.668, 95% CI 1.270–2.191, P = 0.000) with overall survival of PDAC patients. (Table 3) (Fig 1) Furthermore, multivariate analysis also revealed *FGFR1* and *FGF14* as independent prognostic markers for better overall survival in pancreatic cancer patients (Table 3). In addition, we have also evaluated the association of *FGF14* with IGF pathway genes in the validation cohort. *FGF14* inversely correlated with *IGF2BP1*, *IGF2BP2*, *IGF2BP3*, *IGFBP6* and *IGFL2* (**S6 Fig in** S1 File). Intriguingly, these results were consistent with the findings of cohort 1 and 2 of PDAC patients. Of note, inverse correlation of *FGF14* with *IGF2BP1*, *IGF2BP2*, *IGF2BP3*, *IGFBP6* and *IGFL2* was found in all three cohorts of PDAC patients under study.

**Table 3:** Univariate and multivariate analysis of genes for overall survival in cohort 3.

Factors	Univariate Analysis				Multivariable analysis			
	P-value	HR	95.0% CI		P-value	HR	95.0% CI	
			Lower	Upper			Lower	Upper
FGF14	**0.039**	**0.782**	**0.619**	**0.988**	**0.050**	**0.791**	**0.626**	**1.000**
FGFR1	**0.011**	**0.740**	**0.586**	**0.935**	**0.011**	**0.738**	**0.583**	**0.934**
Nodes	**0.000**	**1.668**	**1.270**	**2.191**	**0.000**	**1.707**	**1.299**	**2.243**

Hence, according to our results *FGF14* is the predictor for overall survival in all three PDAC cohorts, while, *FGFR1* is the predictor for overall survival in two out of three PDAC cohorts. Similarly, *FGF14* inversely correlated with oncogenic IGF pathway genes in all three cohorts. Furthermore, common clinicopathological associations with FGF and IGF pathway genes in cohort 1 and 2 are summarized in (**S6 Table in** S1 File).

### Identification of *FGF14* co-expressed genes

**Fig 2:**
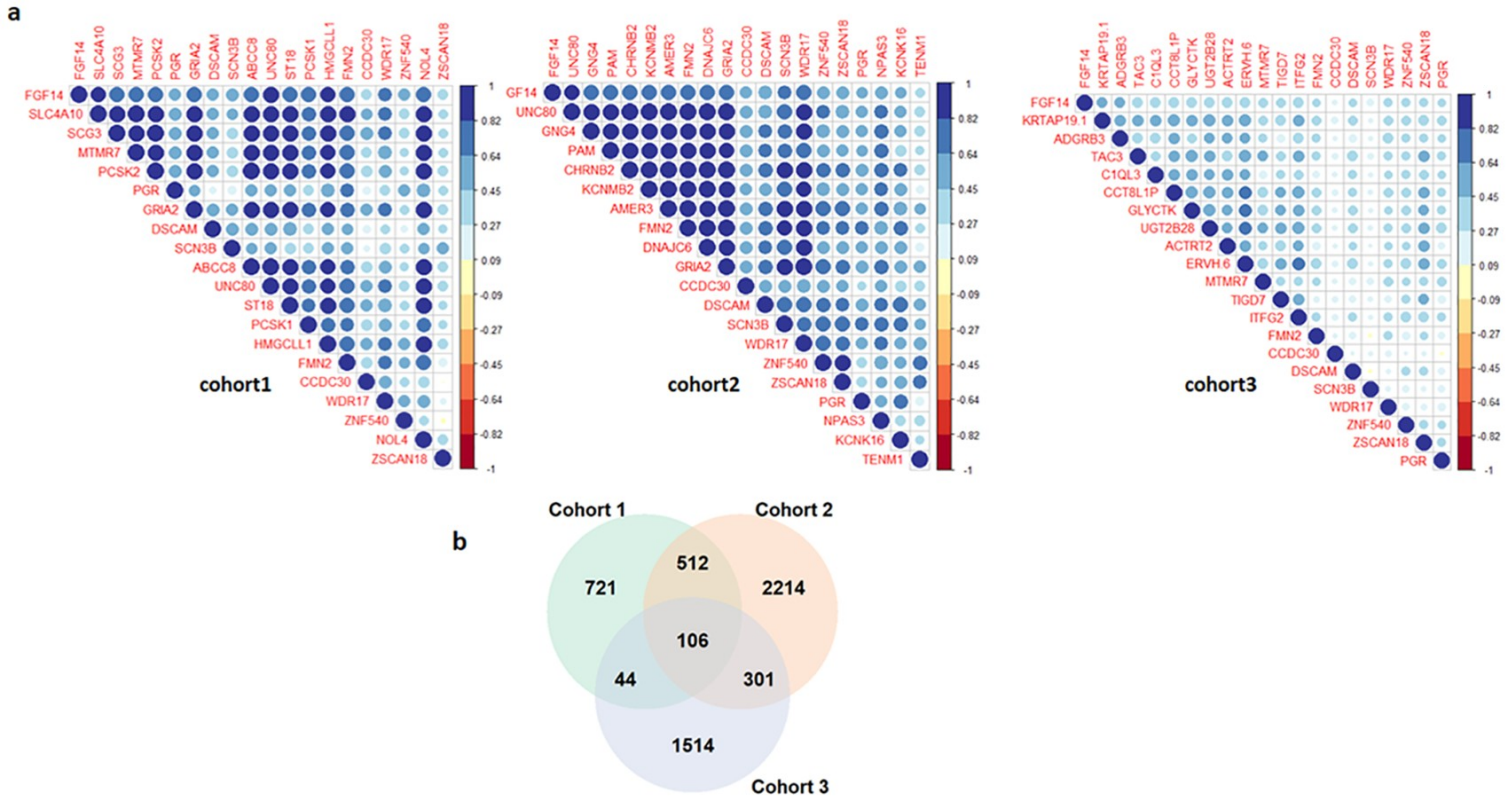
(a) Top positively correlated genes associated with *FGF14* at expression level in three independent cohorts of PDAC patients (b) Venn diagram illustrating top positively correlated genes of *FGF14* at expression level common among the three independent cohorts of PDAC patients.

The correlation of expression profile of *FGF14* gene was examined with the expression profiles of approximately 20,000 available genes across all the three independent cohorts of PDAC patients. The correlation employed on cohort 1, 2 and 3 revealed a large number of genes that are positively correlated with *FGF14* expression in these datasets. Top positively correlated genes are shown in Fig 2a. The common (106) top positively correlated genes of *FGF14* across all the three cohorts were used for further analysis (Fig 2b).

### Functional specification of *FGF14*-coexpressed genes

**Fig 3:**
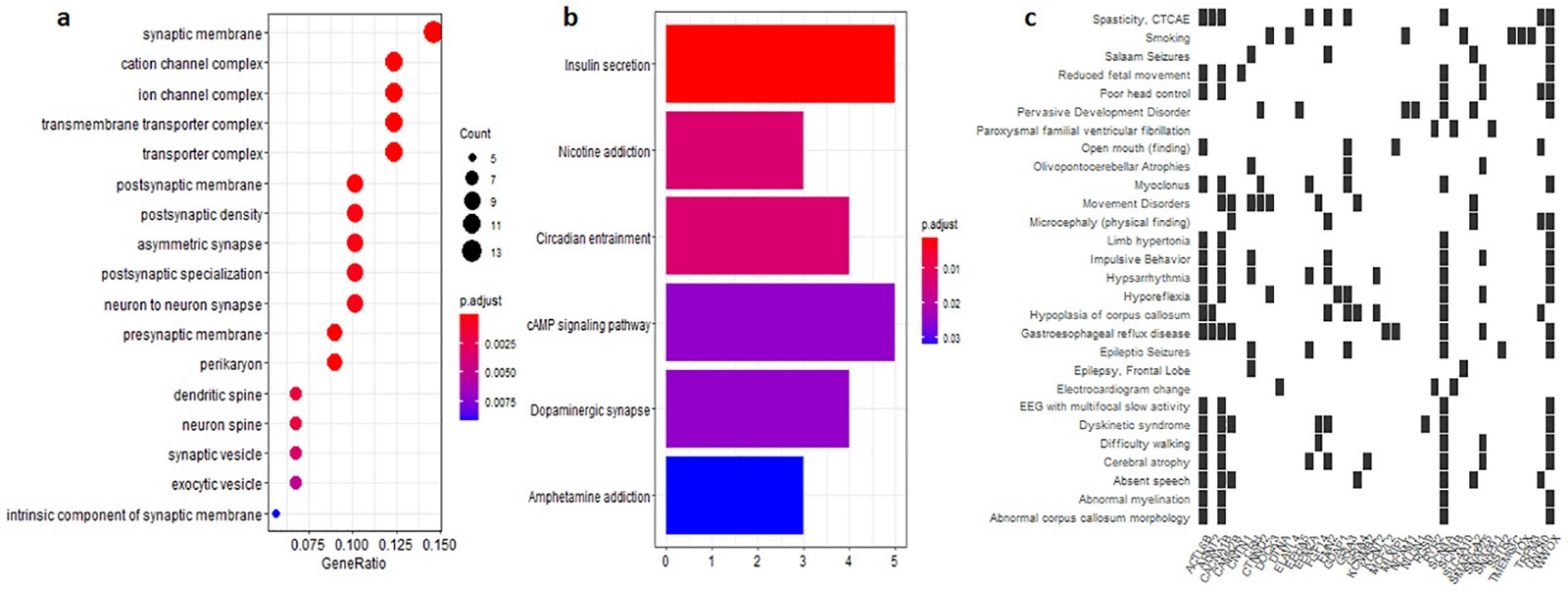
(a) Enriched GO terms among the *FGF14*-specific coexpressed genes common in all three cohorts of PDAC patients (b) Pathway analysis of *FGF14*-specific coexpressed genes common in all three cohorts of PDAC patients (c) Gene-disease associations of *FGF14*-specific coexpressed genes common in all three cohorts of PDAC patients.

According to functional analysis results, co-expressed genes common in all three cohorts clustered into several enriched Gene ontology (GO) terms (Fig 3a) including intrinsic component of synaptic membrane, exocytic vesicles, synaptic vesicles, ion channel complex, transporter complex etc. The GO terms suggest that nerves related membranes and vesicles might play an important role in PDAC. Additionally, the analysis revealed that these *FGF14*-specific coexpressed genes are significantly involved in several Kyoto Encyclopedia of Genes and Genomes (KEGG) pathways (Fig 3b) such as ‘Amphetamine addiction’, ‘Dopaminergic synapse’, ‘cAMP signalling pathway’, ‘Insulin secretion’, etc. Furthermore, different diseases associated with *FGF14*-coexpressed genes were also identified (Fig 3c).

### Prognostic profiling of *FGF14*-specific coexpressed genes

**Fig 4:**
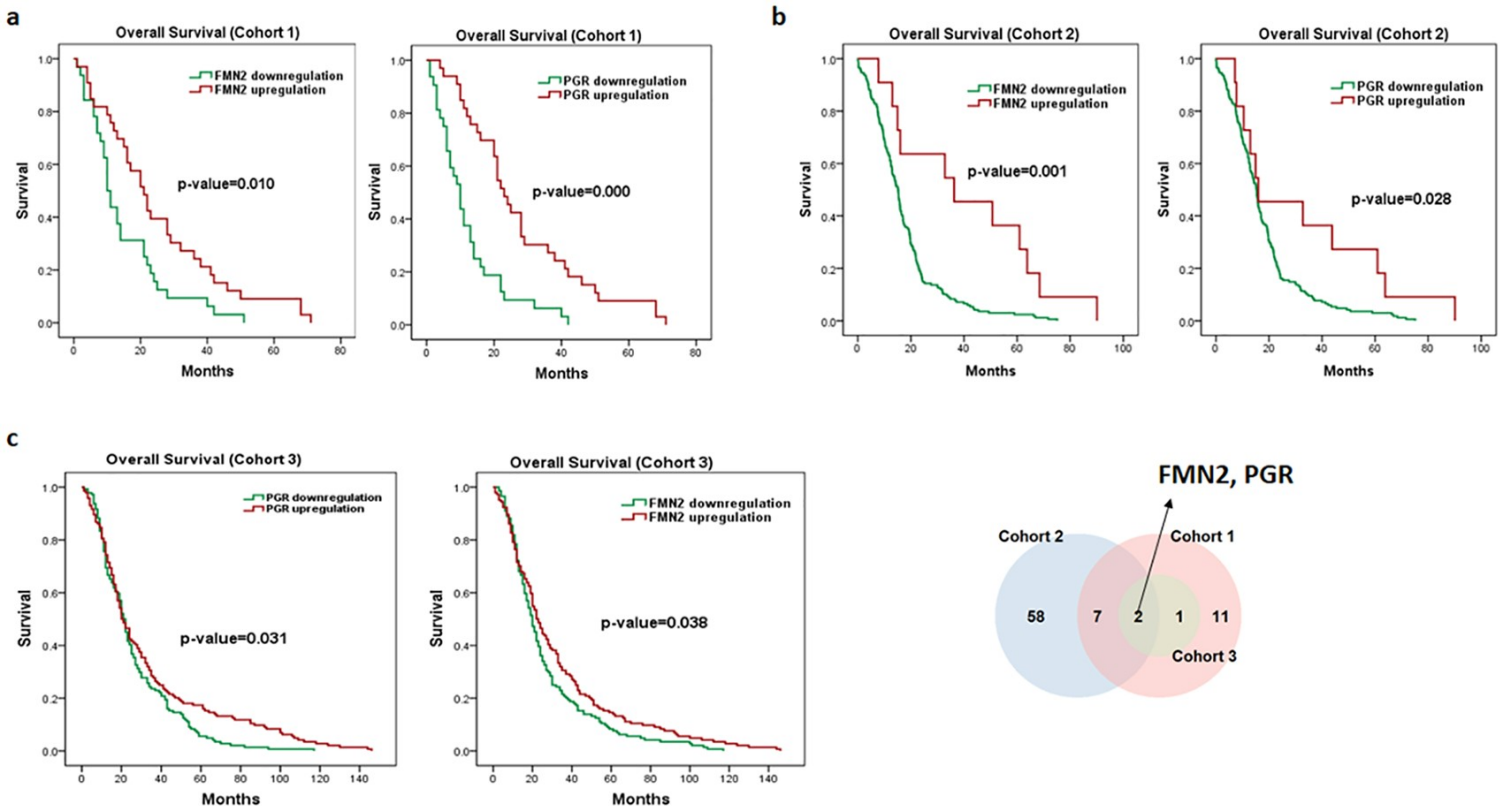
Kaplan–Meier survival analyses of *FMN2* and *PGR* (top common genes of *FGF14*) in PDAC cohorts 1, 2 and 3. Red represents expression ≥ the median, while green represents expression < the median. Overall survival plots of *FMN2*, *PGR* (**a**) in cohort 1. Overall survival plots of *FMN2*, *PGR* (**b**) in cohort 2. Overall survival plots of *FMN2*, *PGR* (**c**) in cohort 3. (* p ≤ 0.05).

According to Kaplan Meier plots for OS, several genes out of 106 *FGF14*-specific coexpressed genes showed significant association with the OS of PDAC patients in cohort 1 and 2 (**S7 and S8 Tables in** S1 File). Predominantly, the overexpression of *CCDC30*, *DSCAM*, *FMN2*, *PGR*, *SCN3B*, *WDR17*, *ZNF540* and *ZSCAN18* from *FGF14*-specific coexpressed genes showed significant association with good OS in cohort 1 and 2 (**S5 Fig in** S1 File). Next the association of these *FGF14*-specific coexpressed genes that correlated with OS in PDAC patients was examined in cohort 3 of PDAC patients. Notably, the overexpression of *FMN2* and *PGR* genes showed association with good OS in all three cohorts of PDAC patients, suggesting the potential to formulate a *FGF14*-specific signature (Fig 4).

## Discussion

For FGF pathway, the downregulation of *FGF9*, *FGF14* and *FGFR1* genes was associated with advanced clinical features, suggesting its potential tumor suppressive role (**S6 Table in** S1 File). Particularly, *FGF14* expression, was identified as an independent prognostic marker in all three PDAC cohorts. To the best of our knowledge this is the first study demonstrating the linkage of *FGF14* dysregulation with PDAC patients. In addition, we suggest that overexpression of *FMN2* and *PGR* is correlated with good OS in PDAC. The main findings of our study suggest that mRNA expression of *FGF14*, *FMN2*, *PGR* and *FGFR1* genes define a potentially new molecular subtype of PDAC.

Earlier, reduced *FGFR1* was reported in multiple cancers including lung, pancreas, and breast cancers [23–25]. In addition, downregulation of *FGF9* was reported to regulate tumorigenesis in lungs cancer [26]. Interestingly, prediction of *FGF14* as a novel biomarker of survival is of immense clinical significance. Previously, the dysregulation of *FGF14* using pancreatic cancer cell lines was reported [27]. Consistently, Liu et al. also proposed that dysregulation of *FGF14* (homologue of *FGF13*) is involved in progression of cervical cancer [28]. Very recently, Tianhong Su and colleagues extensively investigated the role of *FGF14* in colorectal cancer cell lines. Interestingly, they have suggested the tumor suppressor role of *FGF14* in colorectal cancer [29]. Of note, a recent study reported that *FGF12* (the homologue of *FGF14* [30]) have a malignancy- inhibitor effect on pancreatic cancer cell lines [31]. Two most recent studies reported that *FGF14* overexpression leads to tumor suppressive effects in lung adenocarcinoma and nasopharyngeal carcinoma [32, 33]. In line with the previous *in-vitro* and *in-vivo* studies, our *in-silico* data also suggests that inhibitors targeting *FGF14* expression would prove to be a promising therapeutic option against solid tumors.

For IGF pathway, *IGF2BP1*, *IGF2BP2*, *IGF2BP3*, *IGFBP6* and *IGFL2* inversely correlated with *FGF14*. In addition, *IGF2BP2* was found associated with advanced clinical features in PDAC patients. Consistent with previous studies, up-regulation of *IGF2BP2* promoted liver, colorectal and breast tumorigenesis [34–36]. Similarly, studies have also reported the tumor-promoting role of *IGF2BP1*, *IGF2BP3*, *IGFBP6* and *IGFL2* in several cancers such as hepatocellular carcinoma, pancreatic and prostate cancers [37–40]. The potential association of *FGF14* with IGF pathways genes, if validated by additional studies, may serve as a pivotal to understand the existing associations between growth factor pathways that results in pathogenesis of PDAC.

Moreover, we also evaluated the prognostic relevance of *FGF14*-specific coexpressed genes. The top genes *CCDC30*, *DSCAM*, *FMN2*, *PGR*, *SCN3B*, *WDR17*, *ZNF540* and *ZSCAN18* which correlated with *FGF14* were found highly associated with good OS in two cohorts. Overexpression of *FMN2* and *PGR* was correlated with good OS in all three cohorts of PDAC patients, implying their relevance as potential prognostic markers. In a very recent study, findings show that *ZNF540* and *PGR* were among top 267 down-regulated genes in the squamous cell lung cancer tissues compared to the adjacent normal tissues [41]. PGR is reported as a mediator for anti-tumor activities exerted by Tamoxifen drug in ER+-breast cancer tissue [42]. The expression of *FMN2* is predominantly observed in tumor suppressor pathway [43]. Moreover, the dysregulation of *FMN2* is identified in large cohort of colorectal cancer patients, and could serve as early diagnostic marker of colorectal cancer [44]. The current study extensively demonstrated the potential link of *FGF14* overexpression with the better prognosis of PADC patients, followed by *FGF14* based subtyping and strong literature support. This is the first study demonstrating the role of *FGF14* and its associated genes in PDAC, extensively elucidating the underlying molecular mechanism. Therefore, we suggest that *FGF14* based classification is helpful in categorizing PDAC patients into good and poor prognosis groups. Thus, this study can pave way to design *FGF14* specific inhibitors with good efficacy.

Furthermore, *FGFR1* showed promising results in our study along with good correlation profiles with *FGF14* (**S7 Fig in** S1 File). Our results of *FGFR1* expression are in agreement with our recent study in which we established the clinical significance of *FGFR1* through analysis of 313 pancreatic cancer patients along with the cross-validation done through Immunohistochemistry (IHC) of *FGFR1* protein. Consistently, *FGFR1* overexpression was found significantly correlated with the better overall survival in pancreatic cancer patients [45].

Altogether, we conclude that *FGF14* overexpression may be used as prognostic biomarker for better overall survival in PDAC patients. In addition, our data suggests that *FMN2* and *PGR* overexpression can also act as good prognostic marker for the diagnosis of PDAC. In future, preclinical studies are required to establish the therapeutic relevance of *FGF14* in pancreatic cancer. The findings of this study may provide a deeper understanding of molecular mechanism involved in the onset and progression of PDAC.

## Supporting information

S1 File (PDF)

## Abbreviations

CI: Confidence interval
DFS: Disease-free survival
FGF: Fibroblast growth factor
GEO: Gene Expression Omnibus
GO: Gene-ontology
IGF: Insulin growth factor
KEGG: Kyoto Encyclopedia of Genes and Genomes
OS: Overall survival
PDAC: Pancreatic ductal adenocarcinoma
HR: Hazard ratio
RMA: Robust multiarray average
RPKM: Reads per kilobase million
