# Supplementary material for: Genomic relevance of FGF14 and associated genes on the prognosis of pancreatic cancer
Source: PLoS One. 2021 Jun 1;16(6):e0252344. doi: 10.1371/journal.pone.0252344 (PMC8168911; doi:10.1371/journal.pone.0252344)
Supplement: S1 File — (PDF) [file pone.0252344.s001.pdf]

Genomic Relevance of FGF14 and associated genes on the prognosis of Pancreatic Cancer

Aroosha Raja, Muhammad Faraz Arshad Malik, Farhan Haq[#]

COMSATS University, Islamabad, Pakistan.

[#]**Corresponding author**
Farhan Haq, PhD
COMSATS University, Islamabad
Email: farhan.haq@comsats.edu.pk
Tel: +92-334-9540954

S1 Fig. Overall Workflow of the study.

S2 Fig. Kaplan Meier Analysis of genes in cohort 1. FGF14, FGFR1 overexpression was associated with better overall (A) (B) and overexpression of IGF2BP2, IGF2BP3 associated with lower overall survival (C) (D) of PDAC patients. **(*p ≤ 0.05)** Red represents expression ≥ the median, while green represents expression < the median.

S3 Fig. Kaplan Meier Analysis of genes in cohort 2. FGF9, FGF13, FGF14, FGF17, FGFBP3, IGFBPL1 overexpression was associated with better overall survival (A) (B) (C) (D) (E) (H) and overexpression of FGFRL1, IGFBP3, IGFL1 associated with lower overall survival (F) (G) (I) of PDAC patients. **(*p ≤ 0.05)** Red represents expression ≥ the median, while green represents expression < the median.

S4 Fig. Kaplan Meier Analysis of genes in cohort 2. FGF9, FGF12, FGF13, FGF14, FGF17, FGFBP3, IGFBPL1 overexpression was associated with increased DFS (A) (C) (D) (E) (F) (G) (I) and overexpression of FGF10, FGFRL1, IGFBP3, IGFL1 associated with reduced DFS (B) (H) (J) of PDAC patients. **(*p ≤ 0.05)** Red represents expression ≥ the median, while green represents expression < the median.

S5 Fig. Common FGF14-Specific signature genes associated with survival in cohort 1 and 2 **(*p ≤ 0.05)** Red represents expression ≥ the median, while green represents expression < the median.

S6 Fig. Correlation of FGF14 with IGF pathway genes

S7 Fig. Scatterplot showing correlation between FGF14 and FGFR1 in all three study cohorts

S1 Table. Clinicopathological characteristics of 65 PDAC patients of Cohort 1 **(*p ≤ 0.05)**

S2 Table. Clinicopathological characteristics of 179 PDAC patients of Cohort 2 **(*p ≤ 0.05)**

S3 Table. Clinicopathological characteristics of 288 PDAC patients of Cohort 3 **(*p ≤ 0.05)**

S4 Table. Association of genes with clinicopathologic parameters in Cohort 1 **(*p ≤ 0.05)**

S5 Table. Association of genes with clinicopathologic parameters in Cohort 2 **(*p ≤ 0.05)**

S6 Table. Association of common genes with clinicopathologic parameters in Cohort 1 and 2 **(*p ≤ 0.05)**

S7 Table. FGF14-Signature genes associated with survival in cohort 1 **(*p ≤ 0.05)**

S8 Table. FGF14-Signature genes associated with survival in cohort 2 **(*p ≤ 0.05)**

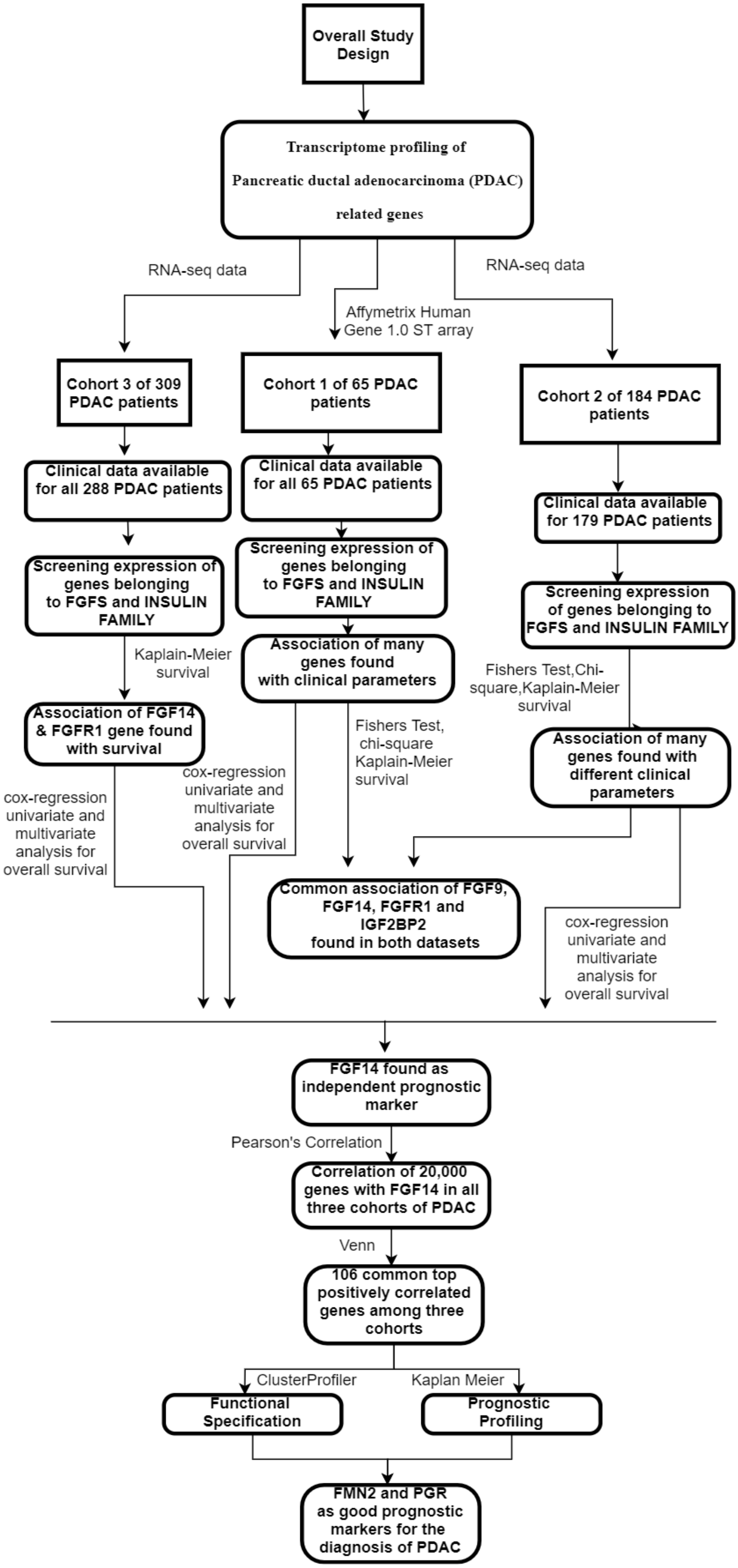
Overall Study Design
Transcriptome profiling of Pancreatic ductal adenocarcinoma (PDAC) related genes
RNA-seq data
Affymetrix Human Gene 1.0 ST array
RNA-seq data
Cohort 3 of 309 PDAC patients
Cohort 1 of 65 PDAC patients
Cohort 2 of 184 PDAC patients
Clinical data available for all 288 PDAC patients
Clinical data available for all 65 PDAC patients
Clinical data available for 179 PDAC patients
Screening expression of genes belonging to FGFS and INSULIN FAMILY
Screening expression of genes belonging to FGFS and INSULIN FAMILY
Screening expression of genes belonging to FGFS and INSULIN FAMILY
Kaplain-Meier survival
Association of many genes found with clinical parameters
Fishers Test,Chi-square,Kaplain-Meier survival
Association of FGF14 & FGFR1 gene found with survival
Fishers Test, chi-square Kaplain-Meier survival
Association of many genes found with different clinical parameters
cox-regression univariate and multivariate analysis for overall survival
cox-regression univariate and multivariate analysis for overall survival
Common association of FGF9, FGF14, FGFR1 and IGF2BP2 found in both datasets
cox-regression univariate and multivariate analysis for overall survival
FGF14 found as independent prognostic marker
Pearson's Correlation
Correlation of 20,000 genes with FGF14 in all three cohorts of PDAC
Venn
106 common top positively correlated genes among three cohorts
ClusterProfiler
Kaplan Meier
Functional Specification
Prognostic Profiling
FMN2 and PGR as good prognostic markers for the diagnosis of PDAC

S1 Fig

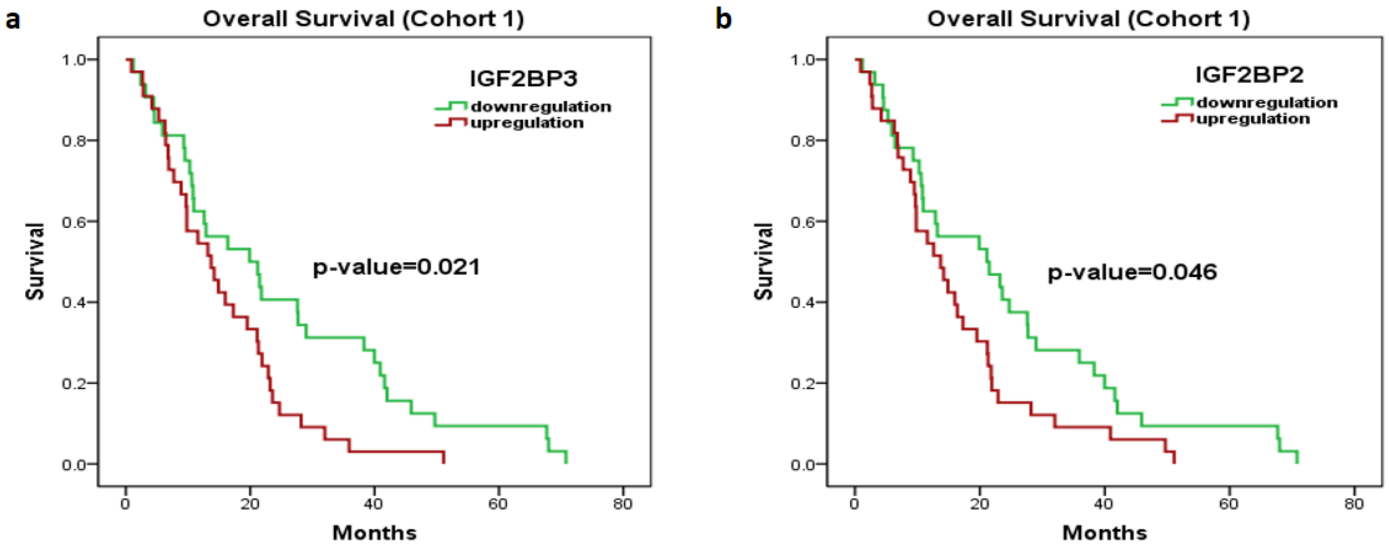
a
Overall Survival (Cohort 1)
IGF2BP3
downregulation
upregulation
p-value=0.021
Survival
Months
b
Overall Survival (Cohort 1)
IGF2BP2
downregulation
upregulation
p-value=0.046
Survival
Months

S2 Fig

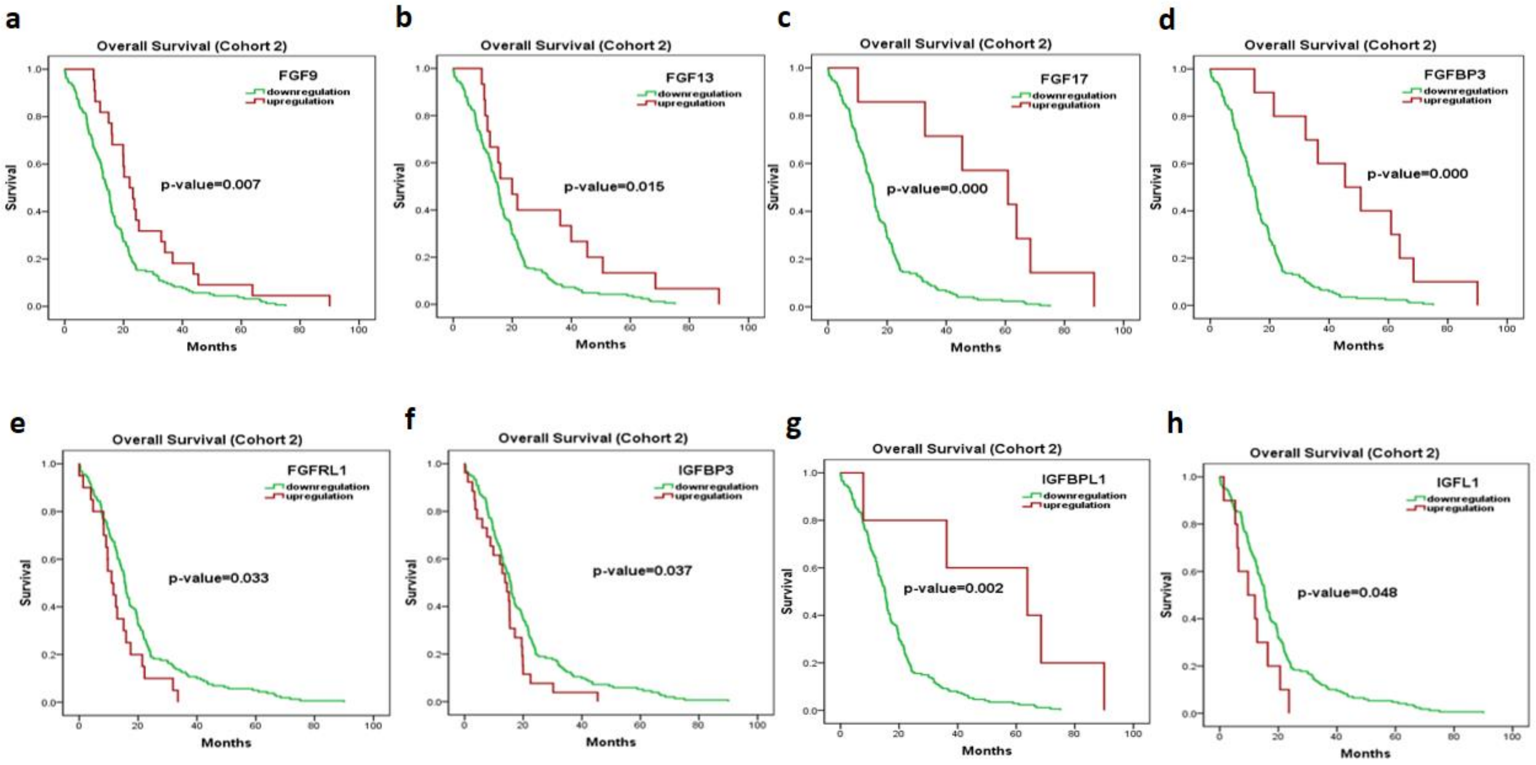
a
Overall Survival (Cohort 2)
FGF9
downregulation
upregulation
p-value=0.007
Survival
Months
b
Overall Survival (Cohort 2)
FGF13
downregulation
upregulation
p-value=0.015
Survival
Months
c
Overall Survival (Cohort 2)
FGF17
downregulation
upregulation
p-value=0.000
Survival
Months
d
Overall Survival (Cohort 2)
FGFBP3
downregulation
upregulation
p-value=0.000
Survival
Months
e
Overall Survival (Cohort 2)
FGFRL1
downregulation
upregulation
p-value=0.033
Survival
Months
f
Overall Survival (Cohort 2)
IGFBP3
downregulation
upregulation
p-value=0.037
Survival
Months
g
Overall Survival (Cohort 2)
IGFBPL1
downregulation
upregulation
p-value=0.002
Survival
Months
h
Overall Survival (Cohort 2)
IGFL1
downregulation
upregulation
p-value=0.048
Survival
Months

S3 Fig

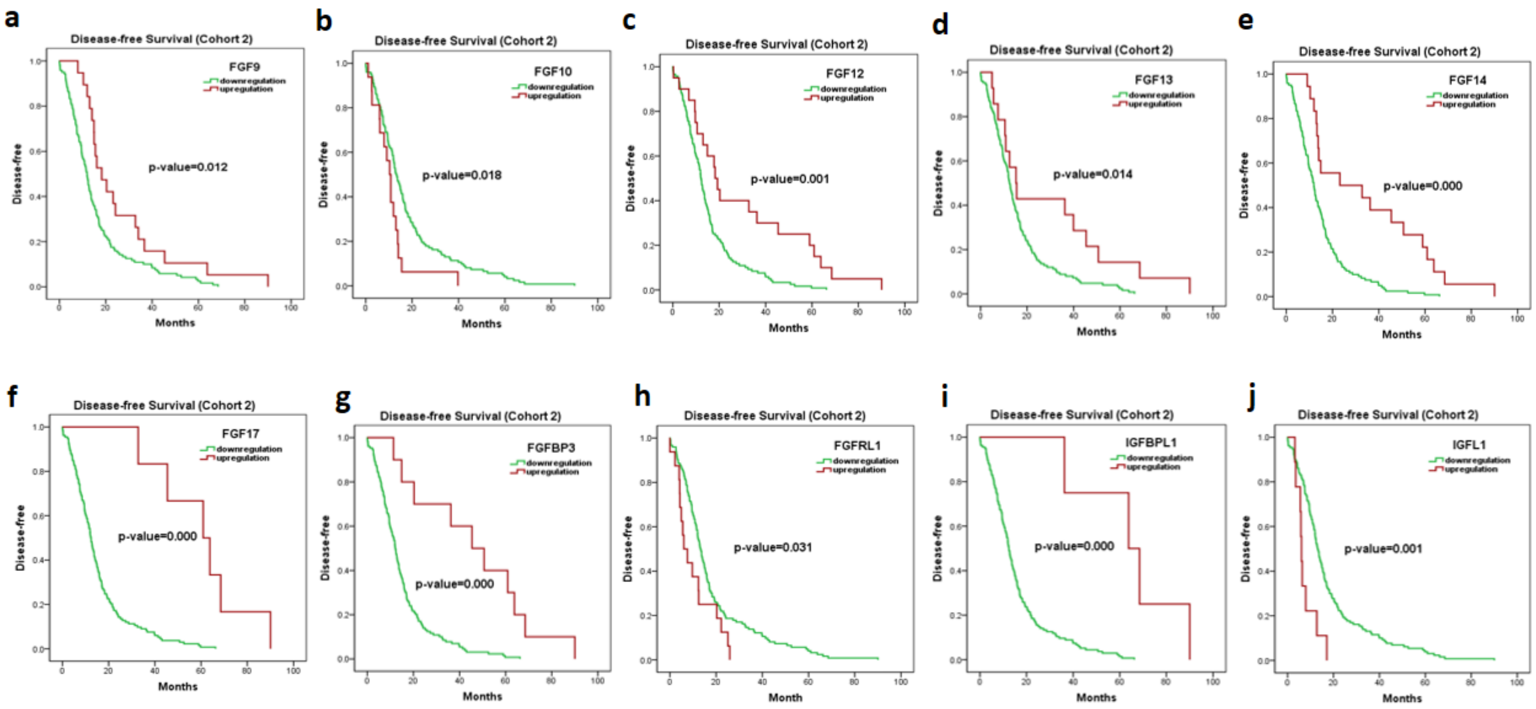
a
Disease-free Survival (Cohort 2)
FGF9
downregulation
upregulation
p-value=0.012
Disease-free
Months
b
Disease-free Survival (Cohort 2)
FGF10
downregulation
upregulation
p-value=0.018
Disease-free
Months
c
Disease-free Survival (Cohort 2)
FGF12
downregulation
upregulation
p-value=0.001
Disease-free
Months
d
Disease-free Survival (Cohort 2)
FGF13
downregulation
upregulation
p-value=0.014
Disease-free
Months
e
Disease-free Survival (Cohort 2)
FGF14
downregulation
upregulation
p-value=0.000
Disease-free
Months
f
Disease-free Survival (Cohort 2)
FGF17
downregulation
upregulation
p-value=0.000
Disease-free
Months
g
Disease-free Survival (Cohort 2)
FGFBP3
downregulation
upregulation
p-value=0.000
Disease-free
Months
h
Disease-free Survival (Cohort 2)
FGFRL1
downregulation
upregulation
p-value=0.031
Disease-free
Month
i
Disease-free Survival (Cohort 2)
IGFBPL1
downregulation
upregulation
p-value=0.000
Disease-free
Months
j
Disease-free Survival (Cohort 2)
IGFL1
downregulation
upregulation
p-value=0.001
Disease-free
Months

S4 Fig

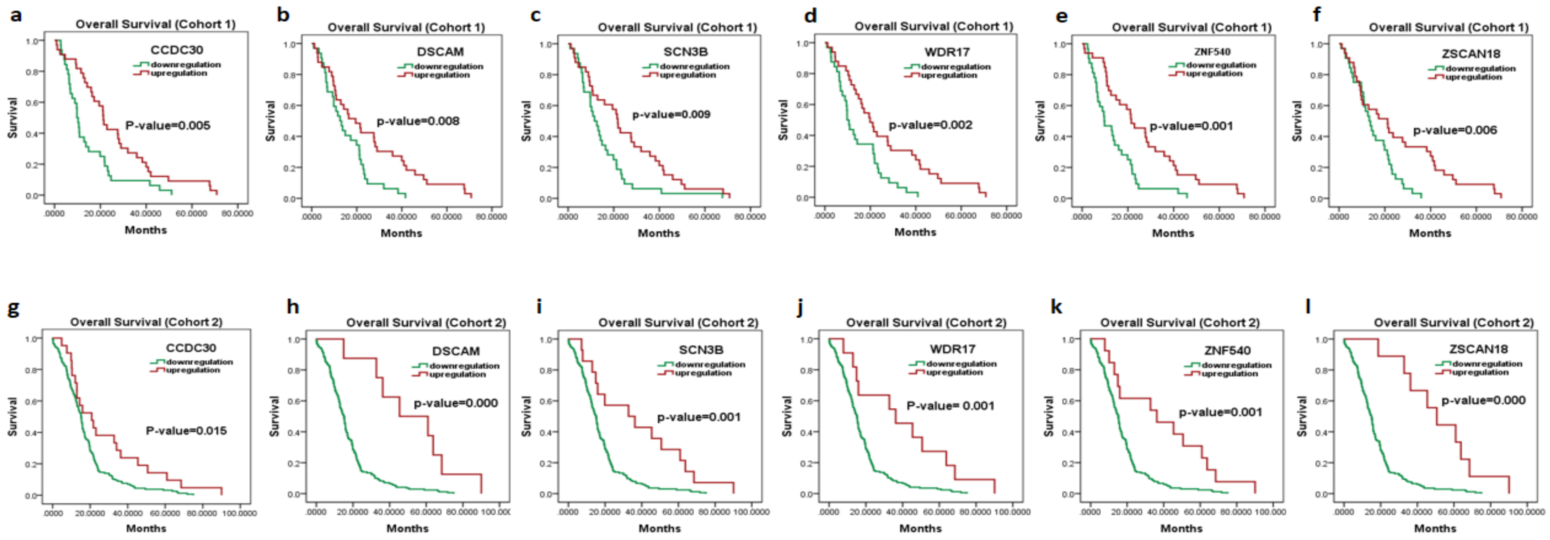
a
Overall Survival (Cohort 1)
CCDC30
downregulation
upregulation
P-value=0.005
b
Overall Survival (Cohort 1)
DSCAM
downregulation
upregulation
p-value=0.008
c
Overall Survival (Cohort 1)
SCN3B
downregulation
upregulation
p-value=0.009
d
Overall Survival (Cohort 1)
WDR17
downregulation
upregulation
p-value=0.002
e
Overall Survival (Cohort 1)
ZNF540
downregulation
upregulation
p-value=0.001
f
Overall Survival (Cohort 1)
ZSCAN18
downregulation
upregulation
p-value=0.006
g
Overall Survival (Cohort 2)
CCDC30
downregulation
upregulation
P-value=0.015
h
Overall Survival (Cohort 2)
DSCAM
downregulation
upregulation
p-value=0.000
i
Overall Survival (Cohort 2)
SCN3B
downregulation
upregulation
p-value=0.001
j
Overall Survival (Cohort 2)
WDR17
downregulation
upregulation
P-value= 0.001
k
Overall Survival (Cohort 2)
ZNF540
downregulation
upregulation
p-value=0.001
l
Overall Survival (Cohort 2)
ZSCAN18
downregulation
upregulation
p-value=0.000
Survival
Months

S5 Fig

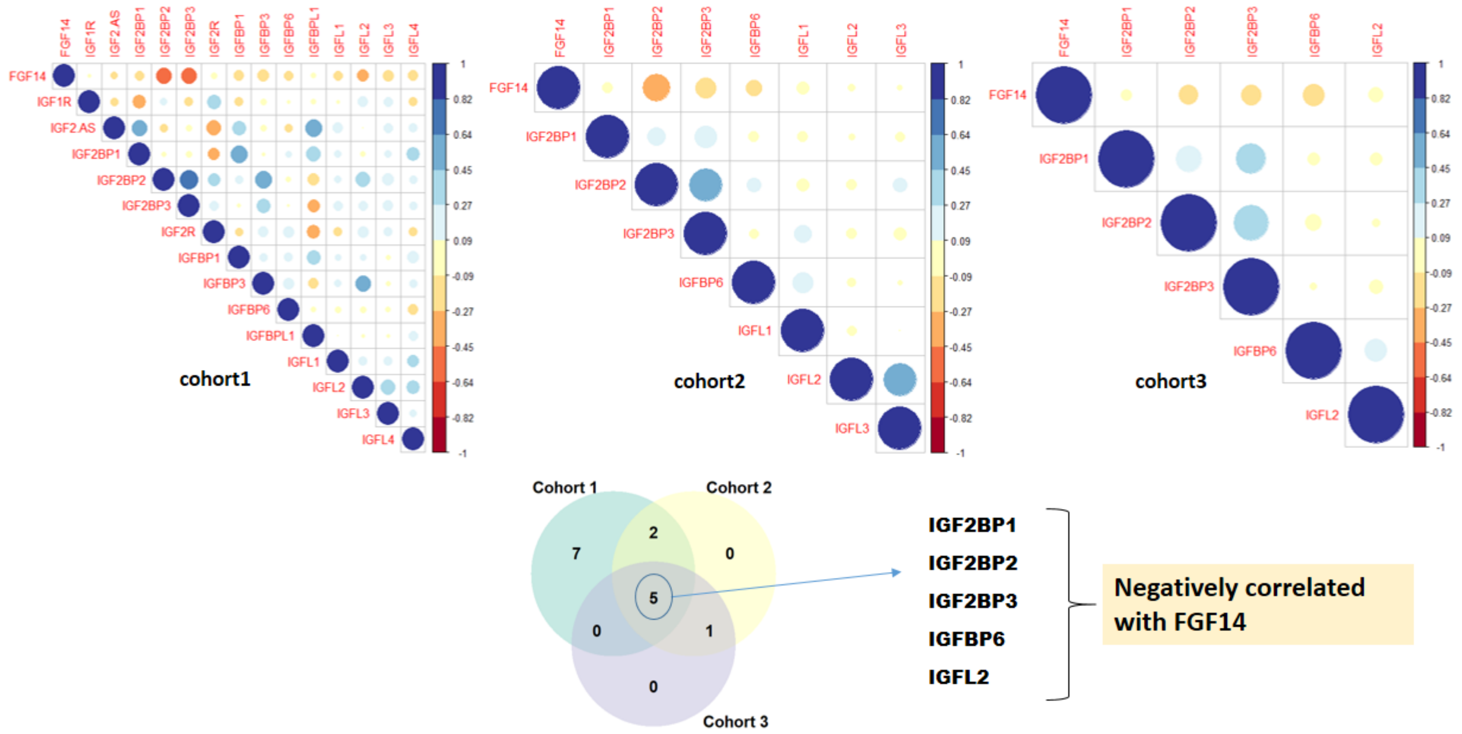
FGF14
IGF1R
IGF2AS
IGF2BP1
IGF2BP2
IGF2BP3
IGF2R
IGFBP1
IGFBP3
IGFBP6
IGFBPL1
IGFL1
IGFL2
IGFL3
IGFL4
cohort1
cohort2
cohort3
1
0.82
0.64
0.45
0.27
0.09
-0.09
-0.27
-0.45
-0.64
-0.82
-1
Cohort 1
Cohort 2
Cohort 3
7
2
0
5
0
1
0
IGF2BP1
IGF2BP2
IGF2BP3
IGFBP6
IGFL2
Negatively correlated with FGF14

S6 Fig

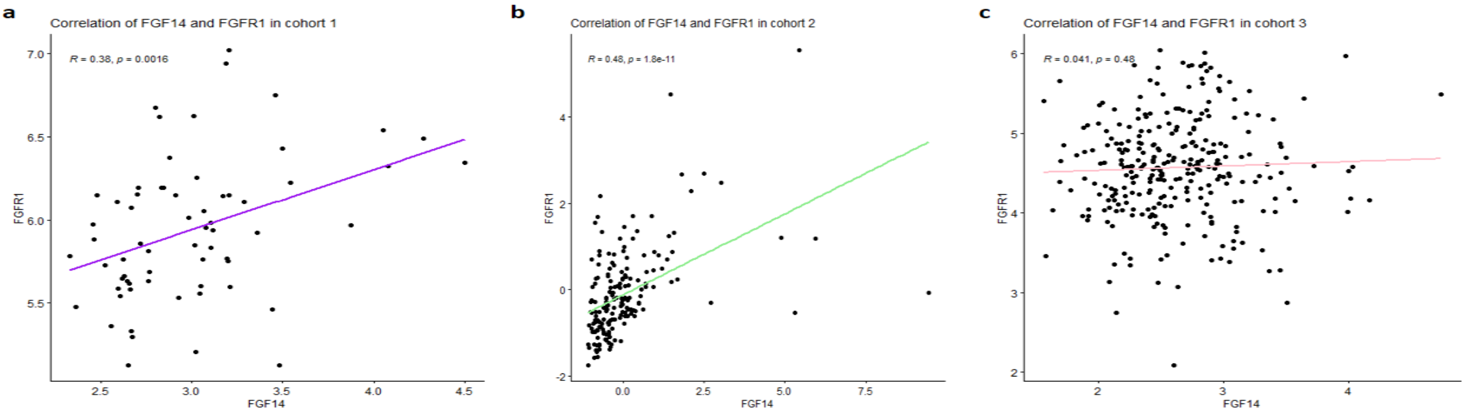
a
Correlation of FGF14 and FGFR1 in cohort 1
R = 0.38, p = 0.0016
FGFR1
FGF14
b
Correlation of FGF14 and FGFR1 in cohort 2
R = 0.48, p = 1.8e-11
FGFR1
FGF14
c
Correlation of FGF14 and FGFR1 in cohort 3
R = 0.041, p = 0.48
FGFR1
FGF14

S7 Fig

S1 Table

Characteristics	No.	Percentage
**Stage**		
Stage I, IA, IB, IIA, IIB	49	75%
Stage III, IV	16	25%
**Grade**		
G1 & G2	34	52%
G3 & G4	30	46%
GX	1	2%
**Dead/Alive**		
Alive	16	25%
Dead	49	75%

S2 Table

Characteristics	No.	Percentage
**Age**		
<=50	24	13%
>50	155	87%
**Gender**		
Male	99	55%
Female	80	45%
**Stage**		
Stage I, IA, IB, IIA, IIB	168	94%
Stage III, IV	8	4%
Discrepancy or (NA)	3	2%
**Tumor (T)-stage**		
T1, T2	31	17%
T3, T4	146	82%
Tx	2	1%
**Nodes**		
N0	50	27%
N1 or greater	124	70%
Nx	5	3%
**Metastasis**		
M0	80	45%
M1 or greater	5	2%
Mx	94	53%
**Residual**		
R0	107	60%
R1 & R2	58	33%
Rx	14	7%
**Dead/Alive**		
Alive	86	48%

Dead	93	52%
**Type-2 Diabetes (T2D)**		
Yes	38	21%
No	109	61%
NA	32	18%
**ALCOHOL**		
Yes	102	57%
No	65	36%
NA	12	7%
**Grade**		
G1 & G2	127	71%
G3 & G4	50	28%
GX	2	1%
**Recurrence**		
DiseaseFree	57	32%
Recurred/Progressed	82	45%
NA	40	23%

S3 Table

Characteristics	No.	Percentage
**Gender**		
Male	166	58%
Female	122	42%
**Nodes**		
N0	76	26%
N1 or greater	212	74%
**Dead/Alive**		
Alive	107	37%
Dead	181	63%
**T-stage**		
T1, T2	51	18%
T3, T4	237	82%

S4 Table

Genes	Classification	Stage	Grade	Alive/Dead
FGF6	FGF Ligands	NA	U	NA
FGF9		NA	U	NA
FGF14		NA	U	NA
FGFR1	FGF Receptors	U	U	U
FGFR1OP2		NA	U	NA
FGFR4		NA	U	NA
IGFBP4	IGF binding protein genes	U	NA	NA
IGF2BP3		NA	U	NA
IGF2BP2		NA	U	NA
IGFL2	Insulin growth factor gene	NA	U	NA
IRS1	Insulin receptor substrate	NA	U	NA

U = upregulated in advance stages, dead;

U = upregulated in early stages, living;

NA = no significant association;

S5 Table

Genes	Classification	Gender	Age	Grade	Stage	Recurrence	Residual	Nodes	Dead/Alive	Diabetes	Alcohol
FGF1	FGF Ligands	NA	NA	NA	NA	NA	NA	U	NA	NA	NA
FGF2		U	NA	NA	NA	U	NA	NA	U	NA	NA
FGF4		NA	U	NA	NA	NA	NA	NA	NA	NA	NA
FGF7		NA	NA	U	NA	NA	NA	NA	NA	U	NA
FGF9		NA	NA	U	NA	NA	NA	U	NA	NA	NA
FGF12		NA	NA	NA	U	U	NA	NA	U	NA	NA
FGF13		NA	NA	NA	NA	U	NA	NA	NA	NA	NA
FGF14		NA	NA	U	U	U	U	U	U	NA	NA
FGF17		NA	NA	NA	U	U	NA	NA	NA	NA	NA
FGF18		NA	NA	U	NA	NA	NA	NA	NA	NA	NA
FGFR1	FGF Receptors	NA	NA	NA	U	U	U	NA	U	NA	NA
FGFR2		NA	NA	NA	NA	NA	NA	NA	NA	U	NA
FGFR4		NA	NA	NA	NA	U	NA	NA	NA	U	NA
FGFBP1	FGFs Binding protein genes	NA	NA	U	NA	NA	NA	NA	NA	NA	NA
FGFBP3		NA	NA	NA	U	U	NA	NA	U	NA	NA
IGF1R	Insulin growth factor receptor	NA	NA	NA	U	NA	NA	NA	NA	NA	NA
IGF1	Insulin growth factor genes	NA	NA	U	NA	NA	NA	NA	NA	NA	NA
IGFL3		U	NA	NA	NA	NA	U	NA	NA	NA	NA
IGFL4		U	NA	NA	NA	U	NA	NA	NA	NA	NA
IGFBP6	IGF binding protein genes	NA	NA	NA	NA	U	NA	NA	NA	NA	NA
IGF2BP2		NA	NA	NA	U	U	NA	NA	NA	NA	NA
IGF2BP3		NA	U	NA	NA	U	NA	NA	NA	NA	NA
IGFBPL1		NA	NA	NA	U	U	NA	NA	NA	NA	NA
INSR	Insulin receptor gene	NA	NA	NA	NA	U	NA	NA	U	NA	NA
INSRR		U	NA	NA	U	NA	NA	NA	NA	NA	NA
IRS2	Insulin receptor substrate	NA	NA	NA	NA	NA	NA	NA	NA	NA	U

U = upregulated in advance stages, yes, >50, female, dead, recurred;
U = upregulated in early stages, no, <=50, male, living, disease-free;
NA = no significant association;

S6 Table

Genes	Classification	Grade	Stage	Nodes	Residual	Dead/Alive	Recurrence
**FGF9**	FGF Ligands	**U**	NA	**U**	NA	NA	NA
**FGF14**	FGF Ligands	**U**	**U**	**U**	**U**	**U**	**U**
**FGFR1**	FGF Receptor	**U**	**U**	NA	**U**	**U**	**U**
**IGF2BP2**	IGF binding protein genes	**U**	**U**	NA	NA	NA	**U**

U = upregulated in advance stages, dead, recurred;
U = upregulated in early stages, living, disease-free;
NA = no significant association;

S7 Table

Genes	P-value	Association
CCDC30	0.005	Overexpression with good OS
DSCAM	0.008	Overexpression with good OS
FGF14	0.001	Overexpression with good OS
FMN2	0.01	Overexpression with good OS
SCN3B	0.009	Overexpression with good OS
WDR17	0.002	Overexpression with good OS
ZNF540	0.001	Overexpression with good OS
ZSCAN18	0.006	Overexpression with good OS
ANKS1B	0.002	Overexpression with good OS
CNTN5	0.005	Overexpression with good OS
KCTD16	0.009	Overexpression with good OS
KIAA0319	0.025	Overexpression with good OS
KRT222	0.029	Overexpression with good OS
NCAM1	0.05	Overexpression with good OS
PCSK1	0.027	Overexpression with good OS
PGR	0	Overexpression with good OS
PPM1E	0.01	Overexpression with good OS
RGN	0.002	Overexpression with good OS
SNAP91	0.007	Overexpression with good OS
TRPM3	0.045	Overexpression with good OS

S8 Table

Genes	P-value	Association
ACTL6B	0.000	Overexpression with good OS
AK9	0.003	Overexpression with good OS
APBB1	0.000	Overexpression with good OS
ARNT2	0.002	Overexpression with good OS
ASTN1	0.001	Overexpression with good OS
ATP8A1	0.019	Overexpression with good OS
ATRNL1	0.001	Overexpression with good OS
CDIP1	0.001	Overexpression with good OS
CAMK2B	0.000	Overexpression with good OS
CCDC30	0.015	Overexpression with good OS
CES4A	0.001	Overexpression with good OS
CLGN	0.000	Overexpression with good OS
CPEB1	0.05	Overexpression with good OS
DDX25	0.002	Overexpression with good OS
DPY19L2	0.001	Overexpression with good OS
DSCAM	0.000	Overexpression with good OS
EFR3B	0.000	Overexpression with good OS
ELAC1	0.000	Overexpression with good OS
ELAVL4	0.024	Overexpression with good OS
EML5	0.004	Overexpression with good OS
EPHA5	0.013	Overexpression with good OS
FAM135B	0.001	Overexpression with good OS
FGF14	0.000	Overexpression with good OS
FMN2	0.001	Overexpression with good OS

GDAP1	0.002	Overexpression with good OS
GRIA3	0.001	Overexpression with good OS
HFM1	0.000	Overexpression with good OS
HYDIN	0.004	Overexpression with good OS
KCNH6	0.000	Overexpression with good OS
KCNMB2	0.002	Overexpression with good OS
KCNT2	0.002	Overexpression with good OS
KIAA1324	0.001	Overexpression with good OS
LRRTM4	0.017	Overexpression with good OS
MCF2L2	0.001	Overexpression with good OS
MLXIPL	0.004	Overexpression with good OS
MTMR7	0.001	Overexpression with good OS
MTUS2	0.028	Overexpression with good OS
NAP1L2	0.001	Overexpression with good OS
NEURL1	0.000	Overexpression with good OS
NLGN1	0.006	Overexpression with good OS
NPAS3	0.007	Overexpression with good OS
TENM1	0.019	Overexpression with good OS
PTPRS	0.006	Overexpression with good OS
RBM11	0.000	Overexpression with good OS
REEP2	0.000	Overexpression with good OS
SCG3	0.001	Overexpression with good OS
SCN3B	0.001	Overexpression with good OS
SLC7A14	0.004	Overexpression with good OS
SNAP25	0.000	Overexpression with good OS
SPTB	0.004	Overexpression with good OS
SSTR2	0.000	Overexpression with good OS
ST18	0.001	Overexpression with good OS
SYT4	0.000	Overexpression with good OS
SYT5	0.000	Overexpression with good OS
TMEM175	0.000	Overexpression with good OS
TMEM63C	0.000	Overexpression with good OS
UNC80	0.000	Overexpression with good OS
VWDE	0.041	Overexpression with good OS
WDR17	0.001	Overexpression with good OS
ZNF540	0.001	Overexpression with good OS
ZSCAN18	0.001	Overexpression with good OS
CTNND2	0.015	Overexpression with good OS
KCNK16	0.005	Overexpression with good OS
LRRTM2	0.014	Overexpression with good OS
PGR	0.028	Overexpression with good OS
RGS9	0.008	Overexpression with good OS
ULK4	0.005	Overexpression with good OS
